# What can be done with today’s budget and demand? Scenarios of rural public transport automation in Mühlwald (South Tyrol)

**DOI:** 10.1007/s12469-023-00333-8

**Published:** 2023-11-27

**Authors:** Alberto Dianin, Michael Gidam, Georg Hauger

**Affiliations:** 1 Faculty of Architecture and Spatial Planning, Vienna University of Technology, Karlsgasse 11, 1040 Vienna, Austria; 2 Eurac Research, Institute for Regional Development, Viale Druso 1, 39100 Bolzano, Italy

**Keywords:** Autonomous vehicles, Rural areas, Scenarios, Public transport, South Tyrol

## Abstract

Rural public transport is typically limited in its coverage, frequency and service period. This is often linked to a low and dispersed demand, which makes the provision of competitive transport services often financially unsustainable. Autonomous vehicles (AVs) might change this condition, allowing for an upgrade of public transport in rural areas. Nevertheless, current studies are mostly focused on urban areas, while the potential for rural applications remains underexplored. This paper contributes to this research line by developing a set of so-called AV scenarios for a potential upgrade of public transport in the rural study area of Mühlwald (South Tyrol, Italy). These scenarios are designed following three core principles. First, line-based and on-demand applications of AVs are not only individually tested, but even combined over space and time in different manners to exploit their synergy. Second, the performances of the scenarios are quantified by assuming the today´s agency cost budget, public-transport demand and peak-hour system capacity to be fixed parameters to comply with. Third, the uncertainties regarding the impacts of AVs on the agency costs are taken into account by defining optimistic, neutral and pessimistic variants of each scenario. Results indicate that the full replacement of current bus lines with a system of on-demand shared taxis might provide the highest performance improvements with the same budget as today, but only with a much bigger fleet. At the same time, the combination of bus lines and on-demand services over time (and space) might provide similarly competitive results while keeping the needed fleet size, service distance and service time relatively low. With this study, policy makers may get insights into the potential improvements of rural public transport that might be initially obtained with different uses of AVs, given a fixed agency cost budget and demand.

## Introduction

In many rural areas, Public Transport (PT) is typically affected by shortcomings such as a limited reach of the network, low frequency of services, or scarce daily coverage (Farrington and Farrington 2005; Hough and Taleqani 2018; Moseley 1979). These issues largely depend on the limited and dispersed transport demand, which often makes rural PT financially unsustainable (ENRD 2016). As a consequence, rural PT mostly meets the mobility demand only during peak hours, while often providing an unsatisfactory standard for the rest of the day (Bernhart et al. 2018). Autonomous Vehicles (AVs) might foster possibilities to change this condition (e.g. Prioleau et al. 2021). For instance, standard fixed-line and -schedule PT could profit from driver cost savings and become more frequent and widespread (Mouratidis and Cobeña Serrano 2021). Yet, alternative transport services, such as shared taxis, could be introduced and provide connections even to the most remote locations (von Mörner 2019). The whole rural population could benefit from such upgrades and especially those groups that typically depend on PT to meet their daily needs (such as students, the elderly, or people with lower income; Milakis and van Wee 2020).

Nevertheless, the automation of rural PT is still an underexplored field of research (e.g. Dianin et al. 2021; Milakis 2019). Indeed, most studies focus on the urban context, where AVs are expected to trigger the most disruptive implications for e.g. congestion, vehicular fleet size, overall mobility degree, or parking space (e.g. Bruck and Soteropoulos 2022; Nahmias-Biran et al. 2019; Viegas 2017). Conversely, rural areas are supposed to be less attractive for innovative shared schemes and more challenging in terms of establishing digital and physical infrastructures (Colonna et al. 2018; Ort et al. 2018; Shaharabani 2018). Despite that, some recent studies have started focusing on the automation of rural PT. For instance, Gühnemann et al. (2019) and Rehrl and Zankl (2018) discuss the possible usages of AVs to fill the first/last-mile gap and complement rural PT. Conversely, Imhof et al. (2020), Schlüter et al. (2021) and von Mörner (2019) show the possible usages and impacts of on-demand AVs (both shared and not shared) when replacing traditional line services. Yet, Sieber et al. (2020) and Zieger and Niessen (2021) test autonomous rail units and the integration between rail lines and autonomous feeder services. Many of these studies focus on standard transport supply concepts like the full replacement of PT lines with on-demand AVs (as Imhof et al. 2020), and they hardly combine different types of AV applications together (Dianin et al. 2022b). Additionally, they rarely deepen the financial feasibility of such concepts for transport providers (e.g. von Mörner 2019).

To contribute to this research line, this study designs and compares a set of scenarios proposing alternative usages of AVs for the upgrade of PT in the rural study area of Mühlwald (South Tyrol, Italy). These scenarios comprise line-based and on-demand services, which are both singularly considered and combined over time and space. All scenarios are designed following a core principle: the performances of the system have to be improved by reinvesting the cost savings generated by automation, and by assuming that the today´s agency cost budget and PT demand are constant. Additionally, the peak-hour capacity supplied at the status quo is set as further standard to meet. In this way, policy makers may get an idea of what kind of improvements could be achieved through transport automation just by using today’s budget, and without considering any increase in PT demand and revenues. The AV scenarios are compared with each other, as well as against scenarios called human-driven (HD), which supply the same services of the AV scenarios but still with the costs related to human driving. This approach allows providing two main contributions. On the one side, we show policy makers what improvements of the transport system might be obtained with AVs following a budget-based approach. On the other side, we show the cost savings that the AV scenarios could allow in comparison with current human-driven services having the same transport performances.

The rest of the paper is structured as follows. Section 2 presents the methodology we use to define, design and compare the AV and HD scenarios. Section 3 applies the methodology to the rural and mountainous study area of Mühlwald in South Tyrol (Italy). Results are finally discussed in Sect. 4, and the limits of the study are highlighted. Section 5 concludes the paper by suggesting possible future directions.

## Methodology

Our methodology follows three main steps, namely: (1) Definition of the scenarios; (2) Design of the scenarios; and (3) Comparison of the scenarios.

### Definition of the scenarios

**Fig. 1:**
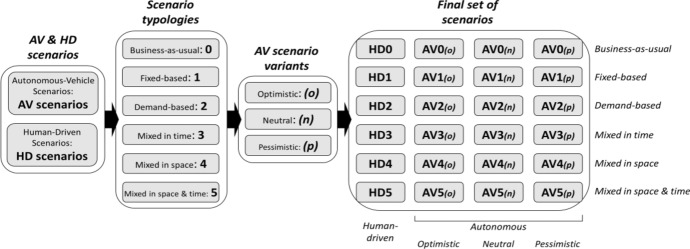
The set of AV and HD scenarios addressed in this study

We distinguish between AV (autonomous-vehicle) and HD (human-driven) scenarios. The former assumes the automation of the rural transport services by means of SAE level 5 vehicles (SAE 2022). The latter assumes that human drivers are still necessary. For both the AV and HD scenarios, we develop six scenario typologies (Fig. 1). Typology 0 (*business-as-usual*) reflects the transport performances of the status quo: HD0 represents the current transport system, while AV0 represents the current transport system but runs with AVs (which is expected to lead to agency cost savings). Typologies 1–5 represent different planning approaches for the improvement of rural transport services. In detail, typology 1 (*fixed-based*) introduces improvements solely focused on line-based transport services. Typology 2 (*demand-based*) hypostatizes the full replacement of line-based services with on-demand shared services. Typology 3 (*mixed in time*) combines line-based and on-demand services by allocating them to different timeframes like peak and off-peak hours. Typology 4 (*mixed in space*) combines line-based and on-demand services by giving them complementary service areas, one acting as trunk and the other as feeder. Typology 5 (*mixed in space & time*) combines the previous two to combine their benefits. For the AV scenarios, we generate three so-called “variants” for each scenario typology (Fig. 1). These variants take into account several uncertainties regarding the impacts of AVs on the agency costs. The first variant is called “optimistic” (*o*) since it considers the most favourable assumptions leading to the highest agency cost savings. The second one is called “neutral” (*n*) since it relies on the most diffused assumptions found in literature. The last one is called “pessimistic” (*p*) because it considers the most negative side effects that could partially offset potential cost savings. According to these criteria, our study comprises 18 AV scenarios (six typologies with three variants each) and six HD scenarios (reflecting the six typologies), as summarised in Fig. 1.

### Design of the scenarios

Following the approach of previous studies as Daganzo (2010), Estrada et al. (2011) and Nocera et al. (2020), the design of the scenarios is focused on three key elements of the PT supply, namely: the transport performances (*TP*) provided by the system; the agency costs (*AC*) incurred by the transport service provider; and the system capacity (*SC*) to be guaranteed during peak hours. In addition, we assume the current PT demand to be constant in the scenarios (thus not influenced by changes in the supply). The estimation of *TP*, *AC* and *SC* follows different principles depending on the considered type of scenario:

- HD0 and AV0 scenarios: *TP*, *AC* and *SC* are calculated at the status quo. AV0 has the same *TP* and *SC* of HD0, but lower *AC* according to the implications of automation (Formula 1).
  1 $$\left\{ {TP_{HD0} = TP_{AV0} ;\;SC_{HD0} = SC_{AV0} ;\;AC_{HD0} > AC_{AV0} } \right\}$$
- AV1-5 scenarios: *TP* is increased for AV1-5 compared to HD0, while *AC* is kept within the status-quo standard, and at least the current peak-hour *SC* is guaranteed (Formula 2).
  2 $$\left\{ {TP_{AV1 - 5} > TP_{HD0} ;\;AC_{AV1 - 5} \le AC_{HD0} ;\;SC_{AV1 - 5} \ge SC_{HD0} } \right\}$$
- HD1-5 scenarios: *TP* and *SC* are the same of the corresponding AV1-5 scenarios, while *AC* is recalculated considering the human-driven costs applied also in HD0 (Formula 3).
  3 $$\left\{ {TP_{HD1 - 5} = TP_{AV1 - 5} ;\;SC_{HD1 - 5} = SC_{AV1 - 5} ;\;AC_{HD1 - 5} > AC_{AV1 - 5} } \right\}$$

**Fig. 2:**
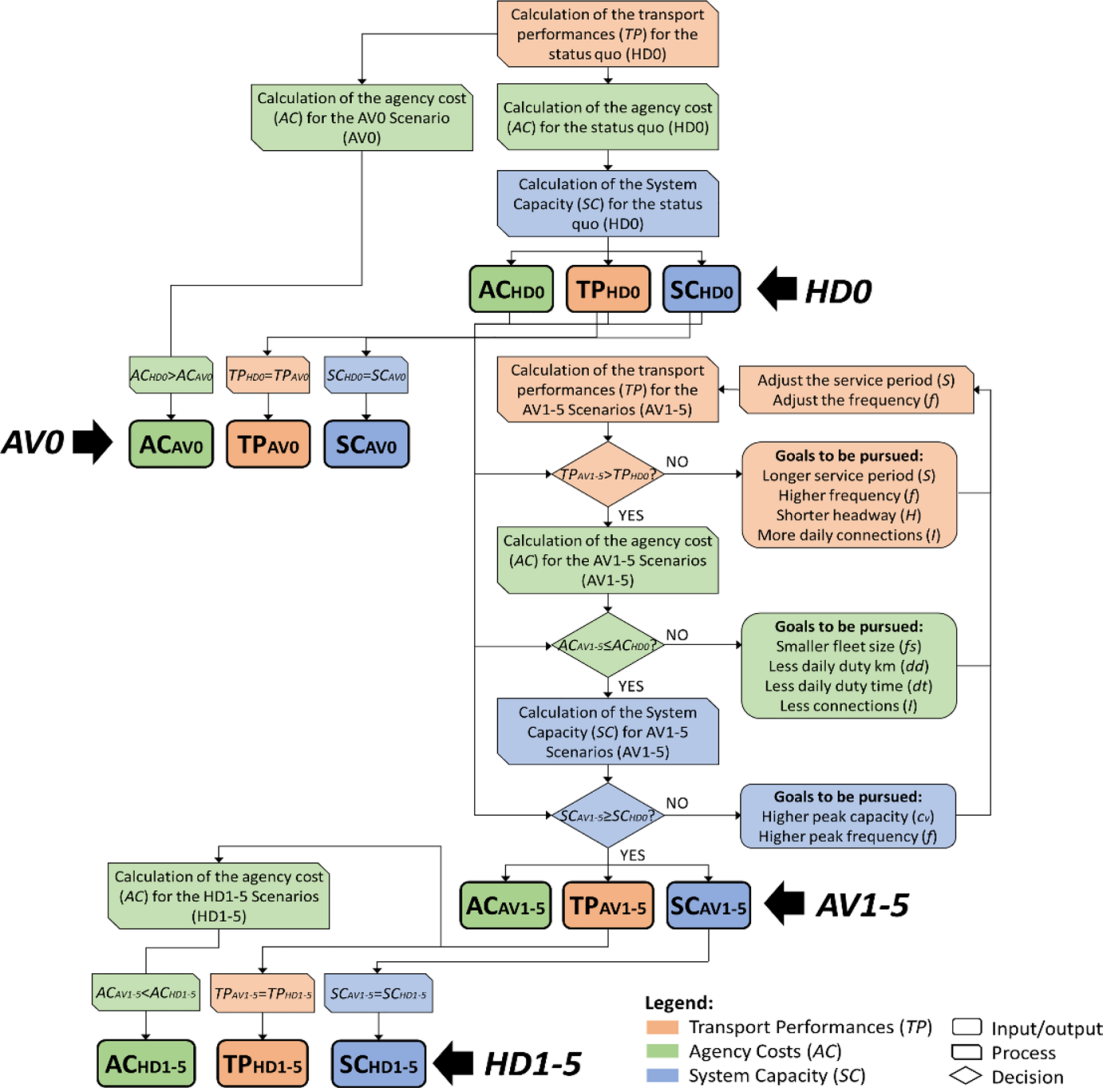
Flow chart for the calculation of *TP*, *AC* and *SC* for the AV and HD scenarios

The next subsections describe how *TP*, *AC* and *SC* are calculated, while Fig. 2 shows the computational process for the scenario design.

#### Transport performances (TP)

*TP* includes seven elements (Formula 4): the service period in hours/day (*S*); the frequency per direction for each service hour in vehicles/hour (*f*); the average daily headway between two consecutive connections in the same direction in minutes ($\bar{H}$); the amount of daily connections in both service directions in vehicles/day (*I*); the fleet size needed to operate the service in vehicles/day (*fs*), the daily duty distance travelled to operate the service in vehicle-km/day (*dd*), and the daily duty time in vehicle-hours/day (*dt*).

Each scenario varies the length of *S* and the value of *f* in each service hour (i.e. independent variables) based on the scenario typology and a constant PT demand. $\bar{H}$, *I*, *fs*, *dd* and *dt* are consequently derived (i.e. dependent variables). For each PT line *x* in the scenarios, the *TP* elements are calculated as follows. *S*_*x*_ is the sum of the service hours (*sh*) supplied per day. *f*_*x*_ is the amount of connections *i* supplied in each service hour (*sh*). $\bar{H}$ is the average of the inverse of the frequencies registered in each service hour (*f*). *I* is the product of the average daily frequency across all service hours (*®f*) and of *S. fs* is derived from the ceiling function ⌈*x*⌉ of the time needed for a full line cycle (*TLC*) and of the maximum hourly frequency across all service hours (*max(f)*). *dd* is product of the one-way length of the line (*L*) and the amount of daily connections (*I*). *dt* is a function of the amount of daily connections (*I*) and the time needed for a full line cycle (*TLC*).

4 $${TP \mathrel\in \left\{ {S,f, \bar{H},I,fs,dd,dt} \right\} \, {\text{with}} \, \bar{H},I,fs,dd,dt = f\left( {S,f} \right) \, {\text{with}} \, \left\{ \begin{gathered} S_{x} = \mathop \sum \limits_{1}^{n} sh_{x} \hfill \\ f_{x} = i_{x} /sh_{x} \hfill \\ \overline{{H_{x} }} = \frac{1}{n}\mathop \sum \limits_{sh = 1}^{n} 60/f_{x} \hfill \\ I_{x} = S_{x} \cdot 2\overline{{f_{x} }} \hfill \\ fs_{x} = \lceil TLC_{x} \cdot {\text{max}}(f_{x} )/60\rceil \hfill \\ dd_{x} = L_{x} \cdot I_{x} \hfill \\ dt_{x} = TLC_{x} /2 \cdot I_{x} /60 \hfill \\ \end{gathered} \right.}$$

#### Agency costs (AC)

According to Ricci (2011), *AC* includes the operational costs for the provision of transport services, it varies for each vehicle category *k* operating in the system, and it includes fixed and variable components. Based on that, we define the daily agency costs (*AC*) as the sum of the cost for each vehicle category *k* operating in the system (*AC*_*k*_). *AC*_*k*_ includes the fixed cost (*FC*), the distance-based variable cost (*DVC*), the time-based variable cost (*TVC*), and the connection-based variable cost (*IVC*; Bösch et al. 2018; Formula 5).^1^

5 $$AC = \mathop \sum \limits_{k = 1}^{n} AC_{k} \; \text{with} \; AC_{k} = FC + DVC + TVC + IVC$$

*FC* (Formula 6) includes the interests (*int*), insurance (*ins*), taxes and levy (*tax*), parking costs (*prk*), highway tolls (*tls*), overheads (*ovr*) and the cleaning costs for line-based services (*cln*_*(lb)*_) that are usually accounted on a vehicular daily basis. It is the product of the unitary fixed daily cost (*fc*) expressed in euro per vehicle-day (€/vd) and of the fleet size (*fs*).

6 $$FC = fc \cdot fs \; \text{with} \; fc = \left\{ {int,ins,tax,prk,tls,ovr,cln_{{\left( {lb} \right)}} } \right\}$$

*DVC*_*d*_ (Formula 7) includes the kilometric depreciation of the vehicle (*dpr*), fuel (*ful*), tyre and lubricant (*tyr*), and maintenance (*mnt*). It is the product of a unitary distance-based daily kilometric cost (*dvc*) expressed in euro per vehicle-kilometre (€/vkm) and of the daily duty distance travelled (*dd*).

7 $$DVC = dvc \cdot dd \; \text{with} \; dvc = \left\{ {dpr,ful,tir,mnt} \right\}$$

*TVC*_*d*_ (Formula 8) comprises mostly the remuneration of the on-board crew (*crw*). Therefore, it is obtained by multiplying a unitary hourly-based cost (*tvc*) expressed in euro/vehicle-hour (€/vh) by the duty time travelled by vehicles in the transport system (*dt*).

8 $$TVC = tvc \cdot dt  \; \text{with} \; tvc = crw$$

*IVC*_*d*_ (Formula 9) comprises the cleaning costs for on-demand services as carsharing and pooling (*cln*_*(od)*_), which are usually accounted on a trip basis, e.g. by performing a vehicle cleaning every n trips made by users (e.g. Bösch et al. 2018). *IVC* is the product of the unitary connection-based cost (*ivc*) expressed in euro/connection (€/v) and of the number of connections daily supplied in the transport system (*I*).

9 $$IVC = ivc \cdot I  \; \text{with} \; ivc = cln_{{\left( {od} \right)}}$$

#### System capacity (SC)

We focus exclusively on the peak-hour system capacity (*SC*^*pk*^) because the PT system has to fully satisfy demand peaks to be a viable alternative to private cars, especially in rural areas. As such, for a given PT line *x* in the designed scenario, *SC* (Formula 10) is calculated as the product of the passenger capacity provided by the fleet during the peak hours (*cv*^*pk*^) expressed in passengers per vehicle (pax/v), and of the line frequency during the peak hours (*f*^*pk*^). Therefore, *SC* is expressed in passengers/hour (pax/h).

10 $$SC_{x}^{pk} = cv_{x}^{pk} \cdot 2f_{x}^{pk}$$

### Comparison of the scenarios

Once all scenarios are designed, they are compared. We carry out three comparisons:

- AV0/HD0 agency cost comparison (Formula 11): The agency cost results of the optimistic (*o*), neutral (*n*) and pessimistic (*p*) variants of AV0 are compared against the current agency costs incurred by HD0. This allows pointing out how much budget the transport provider could save just by switching the current transport services from a human-driven to an automated system.
  11 $$AV0/HD0 = \frac{{AC_{AV0} }}{{AC_{HD0} }} \, {\text{with}} \, AV0 \in \left\{ {AV0_{\left( o \right)} ,AV0_{\left( n \right)} , AV0_{\left( p \right)} } \right\}$$
- HD1-5/HD0 agency cost comparison (Formula 12): The agency costs of the HD1-5 scenarios are compared with those of the status quo (HD0). This shows how much extra budget compared to the status quo would be needed to achieve the transport performances supplied by the pessimistic variant of the AV1-5 scenarios by using traditional human-driven systems.
  12 $$HD1 - 5/HD0 = \frac{{AC_{HD1 - 5} }}{{AC_{HD0} }}$$
- AV1-5/HD0 transport performance comparison (Formula 13): The transport performances of the AV1-5 scenarios are compared with those of the status quo (HD0). Both the optimistic (*o*), neutral (*n*) and pessimistic (*p*) variant are considered. In this way, we highlight the performance benefits that each AV scenario can supply by incurring in the same agency costs as nowadays.
  13 $$AV1 - 5/HD0 = \frac{{TP_{AV1 - 5} }}{{TP_{HD0} }} \; \text{with}\; AV1 - 5 \mathrel \in \left\{ {AV1 - 5_{\left( o \right)} ,AV1 - 5_{\left( n \right)} ,AV1 - 5_{\left( p \right)} } \right\}$$

## Application: the case study of Mühlwald (South Tyrol)

### Study area

**Fig. 3:**
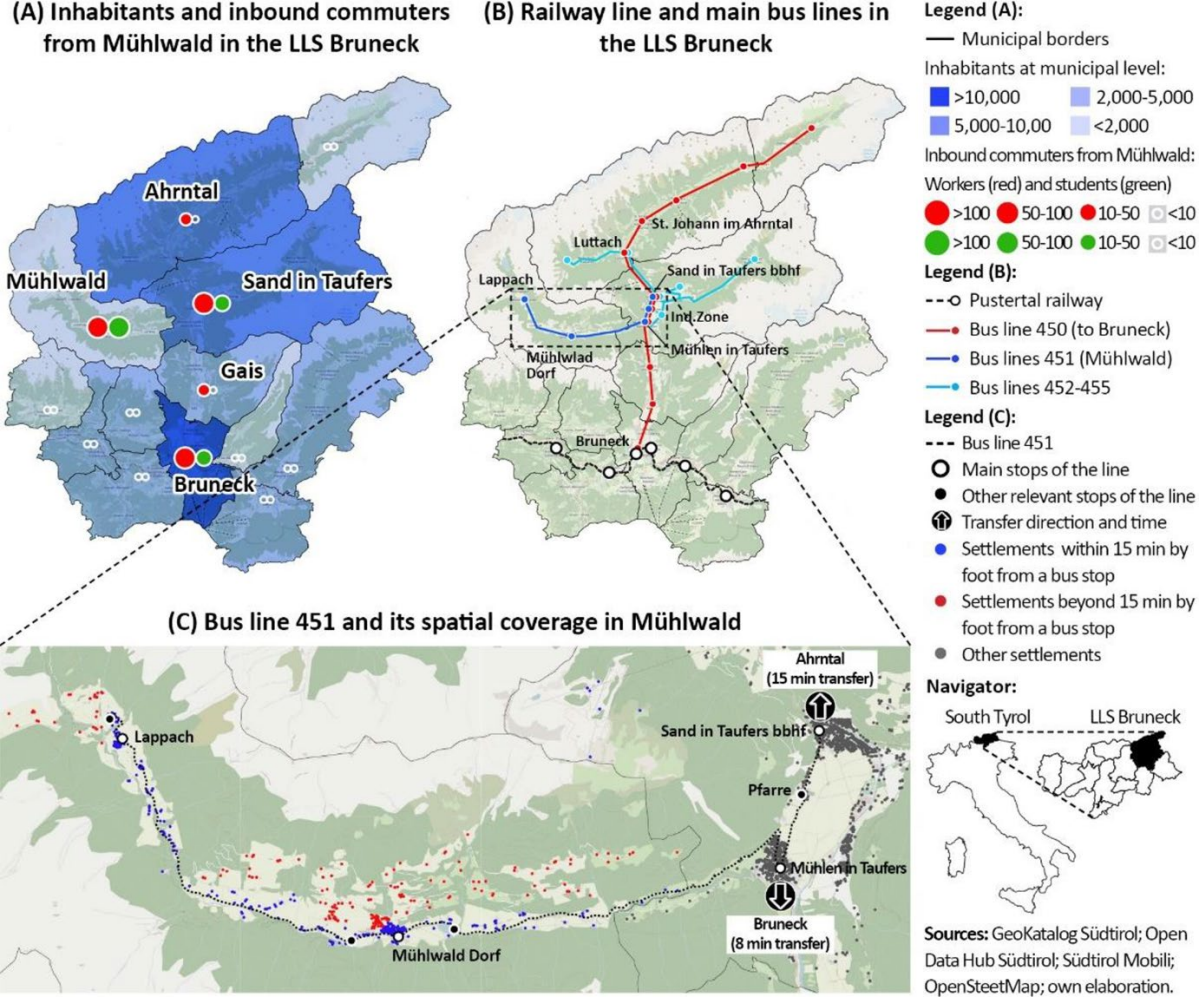
Main territorial and public-transport characteristics of the study area

The municipality of Mühlwald belongs to the so-called Local Labour System^2^ (LLS) of Bruneck (Fig. 3A). The LLS comprises 13 municipalities and about 50,000 inhabitants. Bruneck and Sand in Taufers (16,000 and 5700 inhabitants) are the main centres, while Mühlwald is the least populous municipality (1400 inhabitants). Due to its dimension, Mühlwald highly depends on other municipalities of the LLS Bruneck to access workplaces, middle and high schools and various facilities, thus registering a high share of outbound commuters. 71% of workers and 55% of students in Mühlwald mostly have either Bruneck or Sand in Taufers as destination (ASTAT 2022; ISTAT 2011). As regards the transport supply (Fig. 3B), the LLS Bruneck is crossed by one local railway serving Bruneck. From this node, the main bus line 450 connects the second main town of Sand in Taufers and the neighbouring municipalities to the railway (STA 2022). Additionally, Sand in Taufers is the terminus of five further local bus lines providing connections to smaller agglomerations, including Mühlwald. The bus line 450 provides 60 direct connections to Bruneck per day with a 15-min frequency and it transported more than 4800 passengers/day in 2019 (ASTAT 2022). The other lines offer on average 15 daily connections per direction with an hourly frequency (STA 2022).

Among the minor bus lines cited above, the line 451 serves Mühlwald (Fig. 3C). From west to east, the line serves the villages of Lappach, Mühlwald Dorf, all the settlements located along the main road, and the three built-up areas of Mühlen in Taufers, Pfarre and Sand in Taufers. The northern part of Mühlwald Dorf and the hamlets north of the main road are more than a 15-min walk from the closest bus stop. From the nodes of Mühlen in Taufers and Sand in Taufers, passengers can change from the line 451 to 450, after an average transfer time of 8 min (southbound) and 15 min (northbound). This has negative impacts on the competitiveness of travel time compared to car for several origin–destination pairs (Table 1). Regarding the *TP* indicators listed in the methodology, the line 451 provides 32 daily connections (*I*) – 16 per direction – with an average frequency (*f*) of one connection per hour ($\bar{H}$ = 60 min). Most connections are supplied within a service period (*S*) of 14 h from 6:30 am to 8:30 pm (first and last departure), except for a couple of them in the early morning and late evening. To run the service, one 50-seat bus is daily needed (*fs*), producing 544 vkm/d (*dd*) and 16.0 vh/d (*dt*). The one-way length of the line (*L*) is 17.0 km and the time for a full line cycle is 60 min, including terminal breaks (STA 2022).

**Table 1:** Comparison of PT and car for a set of connections between Mühlwald Dorf and the LLS Bruneck

Origin (Fig. 3B)	Destination (Fig. 3B)	Travel time by PT			Travel time by car	First and last daily departure	
		In-bus time	Transfer time	Total time		From origin to destination	From destination to origin
Mühlwald Dorf	Mühlen in Taufers	9 min	No transfer	9 min	9 min	06:42–20:42	06:03–22:38
	Sand in Taufers Bbhf	13 min	No transfer	13 min	10 min	06:42–20:42	06:03–22:38
	Ind.Zone	12 min	8 min	20 min	11 min	06:42–20:42	06:01–22:30
	Luttach	20 min	15 min	35 min	15 min	06:42–20:42	05:40–22:10
	St. Johann im Ahrntal	25 min	15 min	40 min	20 min	06:42–20:42	05:32–22:02
	Bruneck	34 min	8 min	42 min	24 min	06:42–20:42	05:01–22:01

According to the description above, the bus line 451 presents some challenges that could be addressed through the introduction of AVs (Table 2). These are derived from the desk analysis summarised above, as well as from the consultation with local stakeholders from the municipality of Mühlwald, the Province of South Tyrol, the South Tyrolean transport agency, and the public mobility-development agencies NOI Techpark and Green Mobility. The AV and HD scenarios aim to tackle these challenges.

**Table 2:** Challenges and related goals for the improvement of the bus line 451

Challenges regarding the bus line 451		Goals for the improvements of the bus line 451
1. The frequency is too low to make PT appealing for local travels	→	1. Increase the frequency to make the line 451 more appealing for both local and commuting travels
2. The frequency prevents from making the most of the line 450	→	
3. A transfer of ca 10 min is needed to reach most destinations	→	2. Minimise the transfer time with the trunk line 450 and decrease the overall travel time when possible
4. The travel time by bus is in most cases sensibly longer than by car	→	
5. Before 6:00 and after 20:00 connections are mostly absent	→	3. Broaden the service period
6. The line does not serve the upper part of Mühlwald Dorf	→	4. Provide a solution to reach the inhabitants and amenities currently out of reach
7. The line does not serve the five hamlets of Mühlwald	→	

### Set of scenarios

**Fig. 4:**
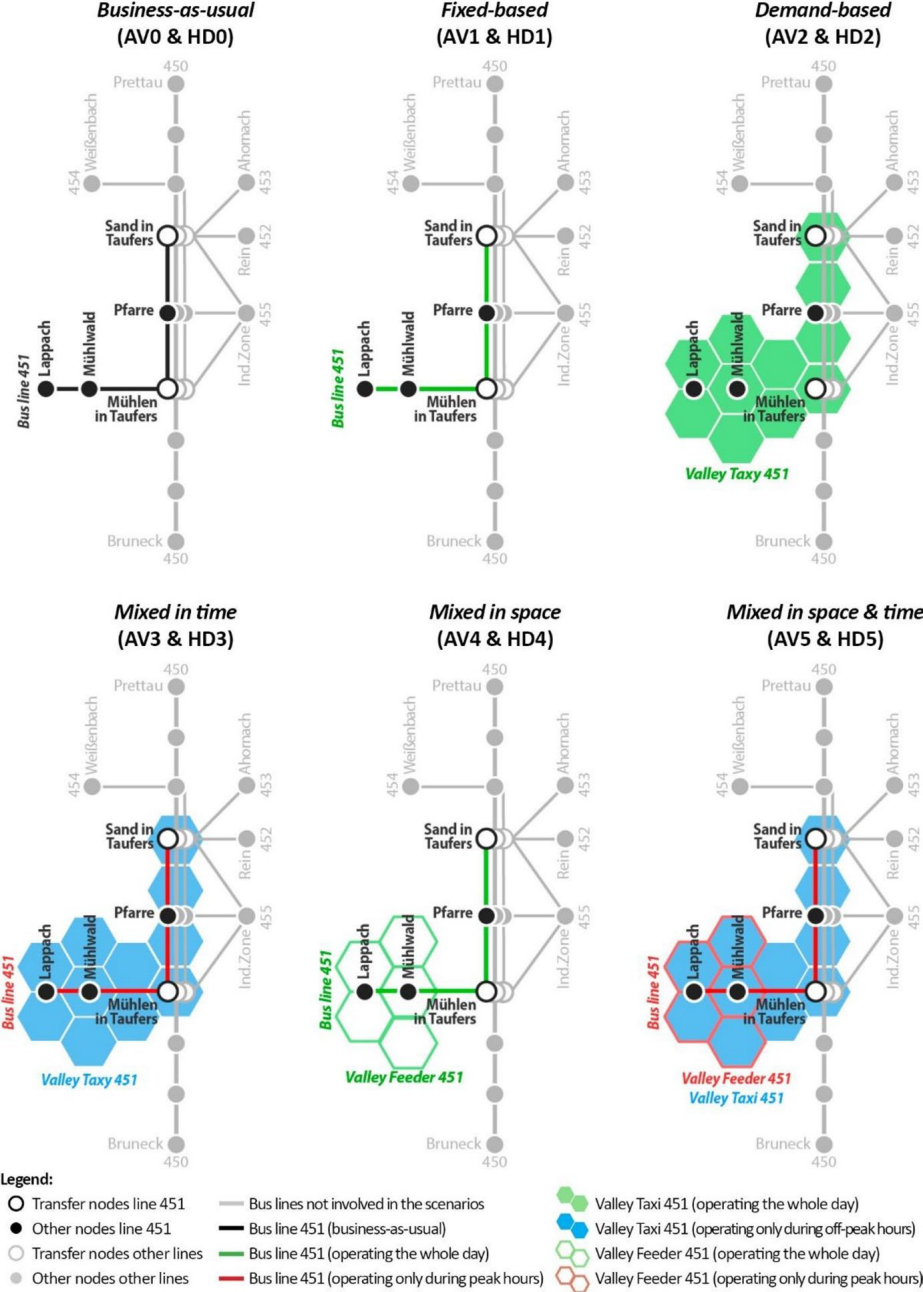
Schematisation of the set of AV and HD scenarios introduced in Mühlwald

To meet the goals, the following AV and HD scenarios are developed (Fig. 4) according to the typologies defined in subsection 2.1 and then designed following the process defined in subsection 2.2:

- Business-as-usual (AV0 & HD0): HD0 represents the transport system at the status quo. Even AV0 assumes the transport performances of the status quo to remain the same. However, the system is served by AVs, which decrease the agency costs needed to operate the system compared to HD0.
- Fixed-based (AV1 & HD1): The fixed schedules, routes and stops of the bus line 451 are kept. However, its competitiveness is improved in three ways. First, the frequency is increased and homogenised throughout the whole service period. Second, the service period is extended and standardised. Third, the coordination of transfers is improved to minimise the transfer time.
- Demand-based (AV2 & HD2): The bus line 451 is replaced by an on-demand service called “Valley taxi 451”. It provides door-to-door connections within Mühlwald, as well as connections between Mühlwald and the three hubs of the line 450 in the neighbouring municipality of Sand in Taufers, i.e. Mühlen in Taufers, Pfarre and Sand in Taufers Bbhf.
- Mixed in time (AV3 & HD3): The bus line 451 is improved as in AV1 and runs through Mühlwald only during peak hours, while the Valley taxi 451 (introduced in AV2) covers the off peaks. Both services provide connections within Mühlwald, as well as between Mühlwald and Sand in Taufers.
- Mixed in space (AV4 & HD4): The bus line 451 is complemented with a so-called “Valley feeder 451”. They cover two complementary parts of the territory: the former links Mühlwald to the neighbouring municipality of Sand in Taufers. The latter connects all the settlements out of the reach of the bus stops, providing local connections to the nodes of the bus line 451.
- Mixed in space & time (AV5 & HD5): The combination of the bus line 451 and Valley feeder 451 proposed in AV4 is active only during peak hours. During the off-peak hours, only a Valley taxi 451 is available and it operates in the same way presented in the demand-based scenario (AV2).

### Transport performances (TP), agency costs (AC), system capacity (SC)

To calculate *TP*, *AC* and *SC* for each scenario, two preliminary steps are needed. First, the agency cost components are estimated at the status quo to define the unitary costs of the HD scenarios. Second, the impacts of AVs on the cost components are quantified for the optimistic, neutral and pessimistic variants.

#### Agency cost components: HD scenarios

The values of the cost components differ among vehicle categories. In this study, three categories are considered, similarly to those proposed by Bösch et al. (2018): a standard 50-seat bus (*k1*), a 20-seat minibus (*k2*) and a 8-seat van (*k3*). For the status quo, the transport service provider of South Tyrol (STA) provides only an overall operation cost figure for the bus line 451 of about 1500 €/d.^3^ Having this data as reference, the values for each cost category listed in the methodology are reconstructed with empirical data and the overall figure provided by STA is used for their validation (Table 3). Specifically, we rely on the empirical data of the Italian National Agency of Public Transport Agencies (ASSTRA) collected from a representative sample of Italian PT agencies, adjusted to the South Tyrolean case and updated to the year 2019, i.e. the last year before the Covid-19 pandemic (ASSTRA 2013).^4^ Specific details regarding the estimation of each cost component are available at Annex 1. Considering these unitary cost components and the *TP* figures described in Subsection 3.1 (*fs* = 1 *k1* vehicle, *dd* = 544 vkm/d, and *dt* = 16.0 vh/d), we obtain an *AC* value of 1,240 €/d*.* This figure is generally coherent with the data provided by STA (17% lower than the official data), confirming the validity of our estimation. Moreover, the cost of the driver accounts for 40.5% of the overall costs (502 €/d), in line with the findings of the report of the national project ReOPEN SPL, reporting an average share of 40.2% among the South Tyrolean bus operators in 2019 (INVITALIA 2019).

**Table 3:** Agency cost components for the HD scenarios

Unitary cost components		Unit of measure	HD scenarios		
			*k1* (50-seat bus)	*k2* (20-seat minibus)	*k3* (8-seat van)
Interests	*int*	€/vd	14.06	3.89	3.29
Insurance	*ins*	€/vd	10.19	2.78	2.22
Taxes and levy	*tax*	€/vd	5.48	2.74	1.64
Parking^a^	*prk*	€/vd	na	na	na
Highway Tolls^*a*^	*tls*	€/vd	na	na	na
Overheads	*ovr*	€/vd	17.37	17.37	17.37
Cleaning (for line-based services)^b^	*cln*_*(lb)*_	€/vd	25.00	17.50	na
Unitary fixed cost	***fc***	**€/vd**	**72.10**	**44.28**	**24.53**
Depreciation	*dpr*	€/vkm	0.62	0.20	0.12
Fuel	*ful*	€/vkm	0.43	0.21	0.11
Tyres and lubricants	*tyr*	€/vkm	0.02	0.02	0.02
Maintenance	*mnt*	€/vkm	0.15	0.08	0.08
Unitary distance-based variable cost	***dvc***	**€/vkm**	**1.22**	**0.52**	**0.33**
Crew remuneration	*crw*	€/vh	31.38	31.38	31.38
Unitary time-based variable cost	***tvc***	**€/vh**	**31.38**	**31.38**	**31.38**
Cleaning (for on-demand services)^c^	*cln*_*(od)*_	€/v	na	na	0.63
Unitary connection-based variable cost	***ivc***	**€/v**	**na**	**na**	**0.63**
Agency Costs for the bus line 451	***AC***	**€/d**	**1,240**	**na**	**na**

^a^The transport provider does not supply services along highways and does not pay any parking fee to the regional public authority

^b^*cln*_*(lb)*_ is not applicable to *k3*, since this vehicle category is used in this study only for on-demand services

^c^*cln*_*(od)*_ is not applicable to *k1* and *k2*, since these vehicle categories are used in this study only for line-based services

#### Agency cost components: AV scenarios

According to Bösch et al. (2018), automation is supposed to influence in different ways various components of the operational costs of transport services (Table 4). In detail:

- Depreciation and interests: The technological requirements of AVs increase the acquisition costs of vehicles, with effects on the kilometric depreciation (*dpr*) and interests (*int*). This is expected to apply especially to small and mediums-size vehicles (+ 20%), while effects on standard busses are more uncertain. Therefore, we assume different impacts on the standard bus acquisition cost for the optimistic (+ 0%), neutral (+ 10%) and pessimistic variant (+ 20%).
- Overheads: Overhead costs (*ovr*) could increase due to the needed remote management of the AV fleet. This should affect especially smaller sized agencies with a limit administrative staff, while larger agencies are expected to introduce such tasks with a smaller effort. Given this uncertainty, we assume no increase for the optimistic variant, + 10% in the neutral one, and + 20% in the pessimistic variant.
- Cleaning: The absence of personnel in form of a driver could trigger irresponsible customer behaviours, with impacts on the cleaning costs for line-based and on-demand services (*cln*_*(lb)*_ and *cln*_*(od)*_). In the case of individual taxis, Bösch et al. (2018) estimate that such costs could account for 29% of the total costs per passenger-kilometre. However, when it comes to regular busses, current cleaning services are supposed to be still adequate. Given these uncertainties, we use three alternative assumptions. In the optimistic variant, *cln*_*(lb)*_ does not change for *k1* and *k2* while *cln*_*(od)*_ increases by 50% for *k3*. In the neutral case, *cln*_*(lb)*_ increases by 15% for *k1* and *k2,* and *cln*_*(od)*_ increases by 75% for *k3.* In the pessimistic case, an increase by 30% in *cln*_*(lb)*_ is assumed for *k1* and *k2*, while it is by 100% for *cln*_*(od)*_ applied to *k3*.
- Insurance, tyres and lubricants, fuel and driver remuneration: Insurance costs (*ins*) decrease by 50% for all vehicle categories thanks to the higher safety guaranteed by AVs. Tyre and lubricant costs (*tyr*) as well as fuel costs (*ful*) decrease by 10% thanks to the smoother driving of AVs. Finally, the salary of the driver (*crw*) is excluded, except for specific cases with on-board operators.

**Table 4:** Agency cost components for the AV scenarios

Unitary cost components		Unit of measure	AV scenarios								
			Optimistic variant			Neutral variant			Pessimistic variant		
			*k1*^a^	*k2*^a^	*k3*^a^	*k1*^a^	*k2*^a^	*k3*^a^	*k1*^a^	*k2*^a^	*k3*^a^
Interests	*int*	€/vd	14.06	4.67	3.95	15.47	4.67	3.95	16.88	4.67	3.95
		AV/HD	+ 0%	+ 20%	+ 20%	+ 10%	+ 20%	+ 20%	+ 20%	+ 20%	+ 20%
Insurance	*ins*	€/vd	5.09	1.39	1.11	5.09	1.39	1.11	5.09	1.39	1.11
		AV/HD	− 50%	− 50%	− 50%	− 50%	− 50%	− 50%	− 50%	− 50%	− 50%
Taxes and levy	*tax*	€/vd	5.48	2.74	1.64	5.48	2.74	1.64	5.48	2.74	1.64
		AV/HD	+ 0%	+ 0%	+ 0%	+ 0%	+ 0%	+ 0%	+ 0%	+ 0%	+ 0%
Parking^b^	*prk*	€/vd	na	na	na	na	na	na	na	na	na
		AV/HD	na	na	na	na	na	na	na	na	na
Highway Tolls^b^	*tls*	€/vd	na	na	na	na	na	na	na	na	na
		AV/HD	na	na	na	na	na	na	na	na	na
Overheads	*ovr*	€/vd	17.37	17.37	17.37	19.11	19.11	19.11	20.85	20.85	20.85
		AV/HD	+ 0%	+ 0%	+ 0%	+ 10%	+ 10%	+ 10%	+ 20%	+ 20%	+ 20%
Cleaning (for line-based services)^c^	*cln*_*(lb)*_	€/vd	25.00	17.50	na	28.75	20.13	na	32.50	22.75	na
		AV/HD	+ 0%	+ 0%	na	+ 15%	+ 15%	na	+ 30%	+ 30%	na
Unitary fixed cost	***fc***	**€/vd**	**67.01**	**43.67**	**24.07**	**73.90**	**48.03**	**25.81**	**80.80**	**52.39**	**27.55**
		**AV/HD**	**− 7%**	**− 1%**	**− 2%**	** + 2%**	** + 8%**	** + 5%**	** + 12%**	** + 18%**	** + 12%**
Depreciation	*dpr*	€/vkm	0.62	0.24	0.14	0.68	0.24	0.14	0.75	0.24	0.14
		AV/HD	+ 0%	+ 20%	+ 20%	+ 10%	+ 20%	+ 20%	+ 20%	+ 20%	+ 20%
Fuel	*ful*	€/vkm	0.39	0.19	0.10	0.39	0.19	0.10	0.39	0.19	0.10
		AV/HD	− 10%	− 10%	− 10%	− 10%	− 10%	− 10%	− 10%	− 10%	− 10%
Tyres and lubricants	*tyr*	€/vkm	0.02	0.02	0.02	0.02	0.02	0.02	0.02	0.02	0.02
		AV/HD	− 10%	− 10%	− 10%	− 10%	− 10%	− 10%	− 10%	− 10%	− 10%
Maintenance	*mnt*	€/vkm	0.15	0.08	0.08	0.15	0.08	0.08	0.15	0.08	0.08
		AV/HD	+ 0%	+ 0%	+ 0%	+ 0%	+ 0%	+ 0%	+ 0%	+ 0%	+ 0%
Unitary distance-based variable cost	***dvc***	**€/vkm**	**1.18**	**0.53**	**0.34**	**1.24**	**0.53**	**0.34**	**1.30**	**0.53**	**0.34**
		**AV/HD**	**− 4%**	** + 3%**	** + 3%**	** + 1%**	** + 3%**	** + 3%**	** + 6%**	** + 3%**	** + 3%**
Crew remuneration	*crw*	€/vh	0	0	0	0	0	0	0	0	0
		AV/HD	− 100%	− 100%	− 100%	− 100%	− 100%	− 100%	− 100%	− 100%	− 100%
Unitary time-based variable cost	***tvc***	**€/vh**	**0**	**0**	**0**	**0**	**0**	**0**	**0**	**0**	**0**
		**AV/HD**	**− 100%**	**− 100%**	**− 100%**	**− 100%**	**− 100%**	**− 100%**	**− 100%**	**− 100%**	**− 100%**
Cleaning (for on-demand services)^d^	*cln*_*(od)*_	€/v	na	na	0.94	na	na	1.09	na	na	1.25
		AV/HD	na	na	+ 50%	na	na	+ 75%	na	na	+ 100%
Unitary connection-based variable cost	***ivc***	**€/v**	**na**	**na**	**0.94**	**na**	**na**	**1.09**	**na**	**na**	**1.25**
		**AV/HD**	**na**	**na**	** + 50%**	**na**	**na**	** + 75%**	**na**	**na**	** + 100%**

^a^*k1*: 50-seat bus; *k2*: 20-seat minibus; *k3*: 8-seat van

^b^The transport provider does not supply services along highways and does not pay any parking fee to the regional public authority. We assume this condition to remain the same also after the introduction of AVs in the system

^c^*cln*_*(lb)*_ is not applicable to *k3*, since this vehicle category is used in this study only for on-demand services

^d^*cln*_*(od)*_ is not applicable to *k1* and *k2*, since these vehicle categories are used in this study only for line-based services

#### TP, AC and SC figures

**Fig. 5:**
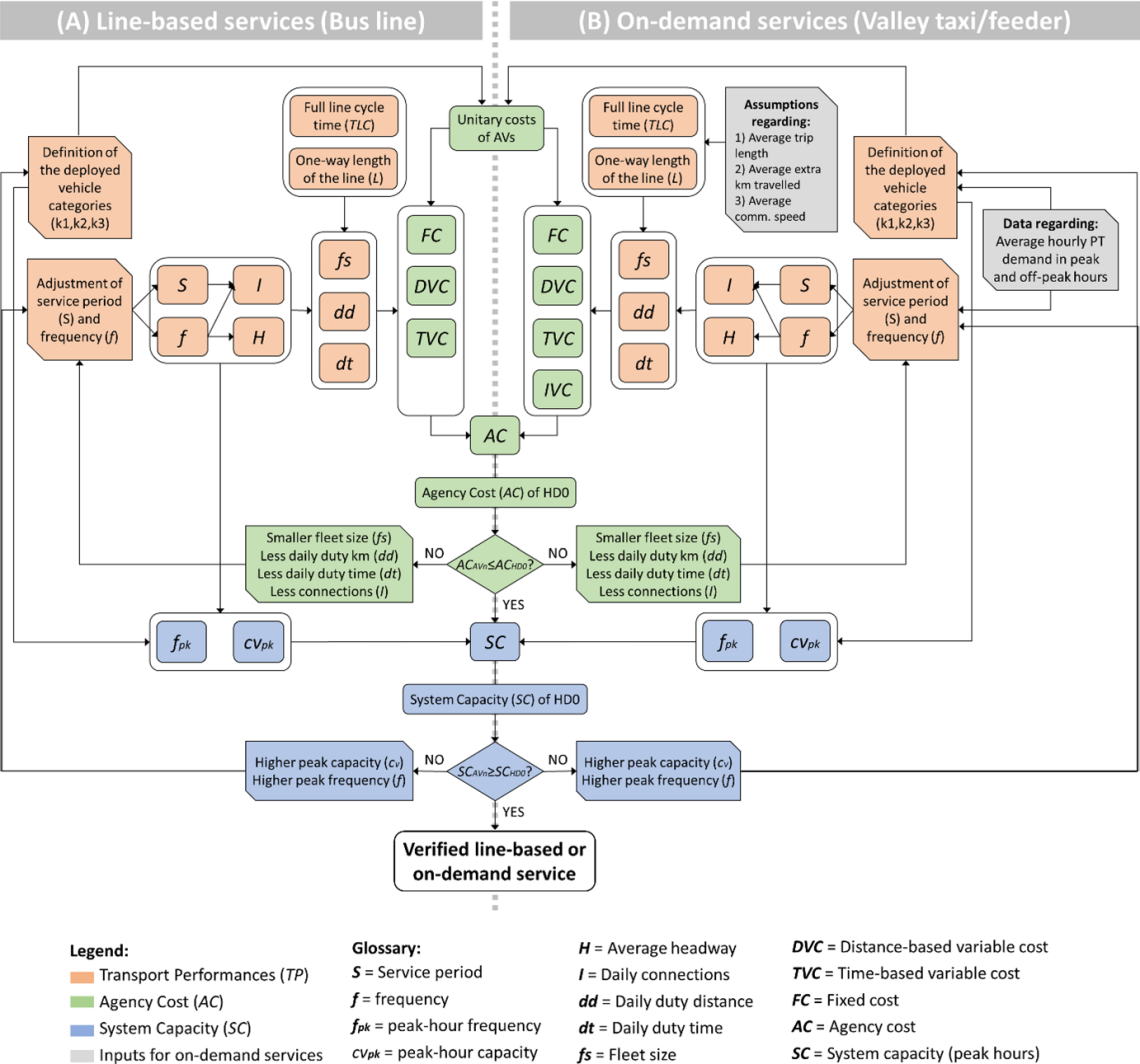
Flow chart of the calculation of *TP*, *AC* and *SC* in the case of (**A**) line-based and (**B**) on-demand services

According to the flow chart presented in Fig. 2 and the cost components estimated above, the *TP*, *AC* and *SC* figures of the scenarios are calculated. Some specifications regarding their computation are necessary, by distinguishing between line-based^5^ and on-demand^6^ services (as summarised in Fig. 5).

Line-based services (Fig. 5A): To improve *TP* for the line-based services (i.e. the bus line 451), we set an increased service period (*S*) and frequency for each service hour (*f*) according to the observed scenario typology. We consequently obtain the reduced average headway on a daily basis ($\bar{H}$) and the increased amount of daily connections (*I*). Based on *S*, *f*, $\bar{H}$ and *I*, as well as on the given one-way length of the line (*L*)^7^ and the time needed for a full line cycle (*TLC*),^8^ we obtain the fleet size (*fs*), daily duty distance (*dd*), and daily duty time (*dt*). We calculate then *AC* considering the unitary costs applied in each scenario. Finally, we calculate *SC* based on the average hourly capacity of the vehicles deployed along the line during the peak hours (*cv*^*pk*^)^9^ and the set peak-hour frequency. If the improved *TP* components require an *AC* value over the current budget or produce a *SC* value under the current standard, they are adjusted until they allow fulfilling all the set requirements. Annex 2 illustrates an exemplificative computation carried out following this process (bus line 451 in scenario AV1_(o)_).

On-demand services (Fig. 5B): For the on-demand services (Valley taxi 451 and Valley feeder 451), the key requirement is serving the current PT demand in the service area. The South Tyrolean transport agency has provided us with data on the hourly PT demand for the line 451.^10^ Based on that, we have derived the average hourly demand in peak and off-peak hours.^11^ Based on the hourly demand and the capacity of the vehicles deployed for the on-demand services,^12^ the minimum amount of connections to provide in each service hour (*f*) and the minimum length of the service period (*S*) are identified. The actual *S* and *f* are set accordingly, and the amount of daily connections (*I*), as well as the average headway on a daily basis ($\bar{H}$) are consequently derived. Afterwards, the daily duty distance and time and the needed fleet size (*dd*, *dt* and *fs*) are estimated similarly to the process followed for line-based services. However, in this case, we rely on assumptions regarding the expected one-way length of the on-demand connections (*L*)^13^ and the time needed for a full on-demand line cycle (*TLC*).^14^ Even the cleaning costs related to the amount of daily provided on-demand connections (*IVC*) are calculated and *AC* is estimated. *SC* is calculated with the same approach adopted for the line-based services.^15^ Annex 3 illustrates an exemplificative computation carried out following this process (Valley feeder 451 in scenario AV4_(o)_).

Table 5 summarises the *TP*, *AC* and *SC* results for all the scenarios. A symbolic colour and shade is assigned to each scenario typology and variant. Results are broken down by each transport service included in the scenarios represented in Fig. 4, namely the Bus line 451, the Valley taxi 451 and the Valley feeder 451. Moreover, the vehicle categories used for each service (*k1*, *k2*, *k3*) and their operating period (whole day; peak hours; off-peak hours) are specified. For the transport services combining more vehicle categories (marked with an asterisk in Table 5), Annex 4 provides further details with disaggregated results. The results reported below are compared in the next subsection and discussed afterwards.

**Table 5:**
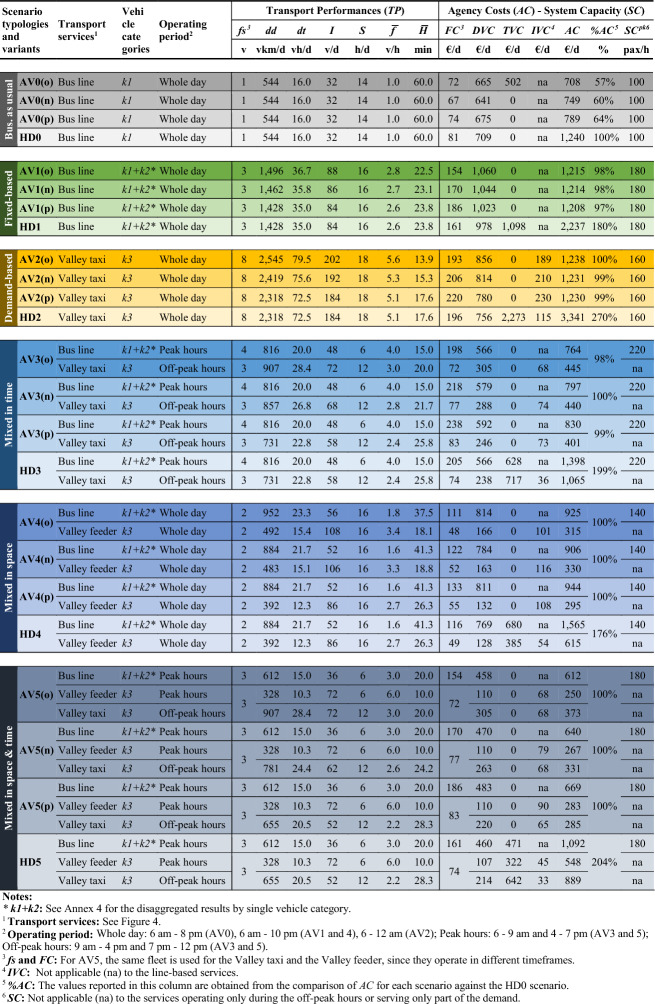
*TP*, *AC* and *SC* figures of the AV and HD scenarios

### Comparison of the scenarios

#### Agency cost comparison

**Fig. 6:**
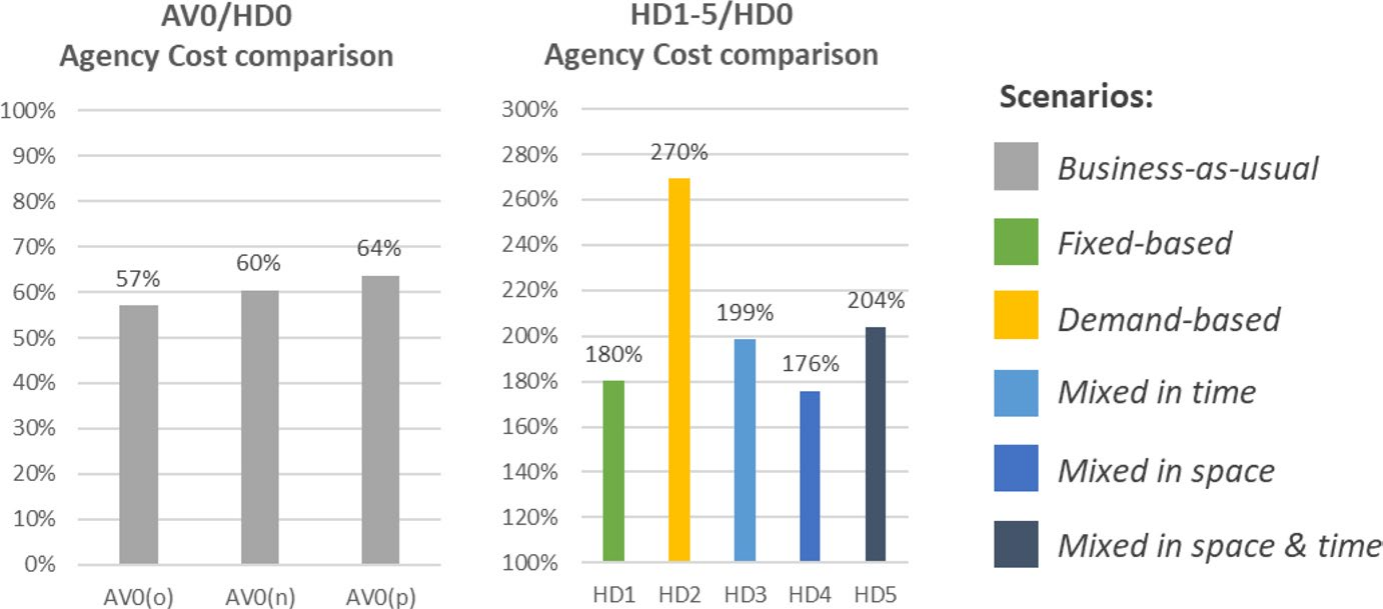
Comparison of the agency cost results: AV0 against HD0 (left); HD1-5 against HD0 (right)

The comparison between the AV0 and HD0 scenario (Fig. 6, left side) shows a clear decrease in operational costs for the transport agency when introducing AV technology, with *AC* savings ranging between 43 and 36% of the current agency costs depending on the considered variant. Regarding AV1-5, these do not aim for lowering agency costs, but for improving the transport performances with the same budget as of now. Figure 6 (right side) shows how much the same AV scenarios would cost in comparison with the status quo (HD0) if the vehicles were operated by a human driver (i.e. HD1-5). To interpret the result, it is fundamental to observe the amount of on-demand services supplied in the scenarios. HD1 and HD4 are the scenarios with the lowest amount of service kilometres and hours supplied by on-demand services (none for HD0; 392 km and 12.3 h for HD4). Accordingly, they registered the lowest increase in *AC* compared to the status quo (+ 80% for HD1 and + 76% for HD4). HD3 and 5 have a midrange amount of on-demand service kilometres and hours (731 km and 22.8 h for HD3; 984 km and 30.8 h for HD5), and even their increase in *AC* is in the midrange (between + 99% and + 104%). Finally, HD2 is the scenario with the most radical adoption of on-demand services (184 daily connections producing 2,318 service km and 72.5 service hours) and it is also that one showing the highest cost increase (+ 170%).

#### Transport performance comparison

**Fig. 7:**
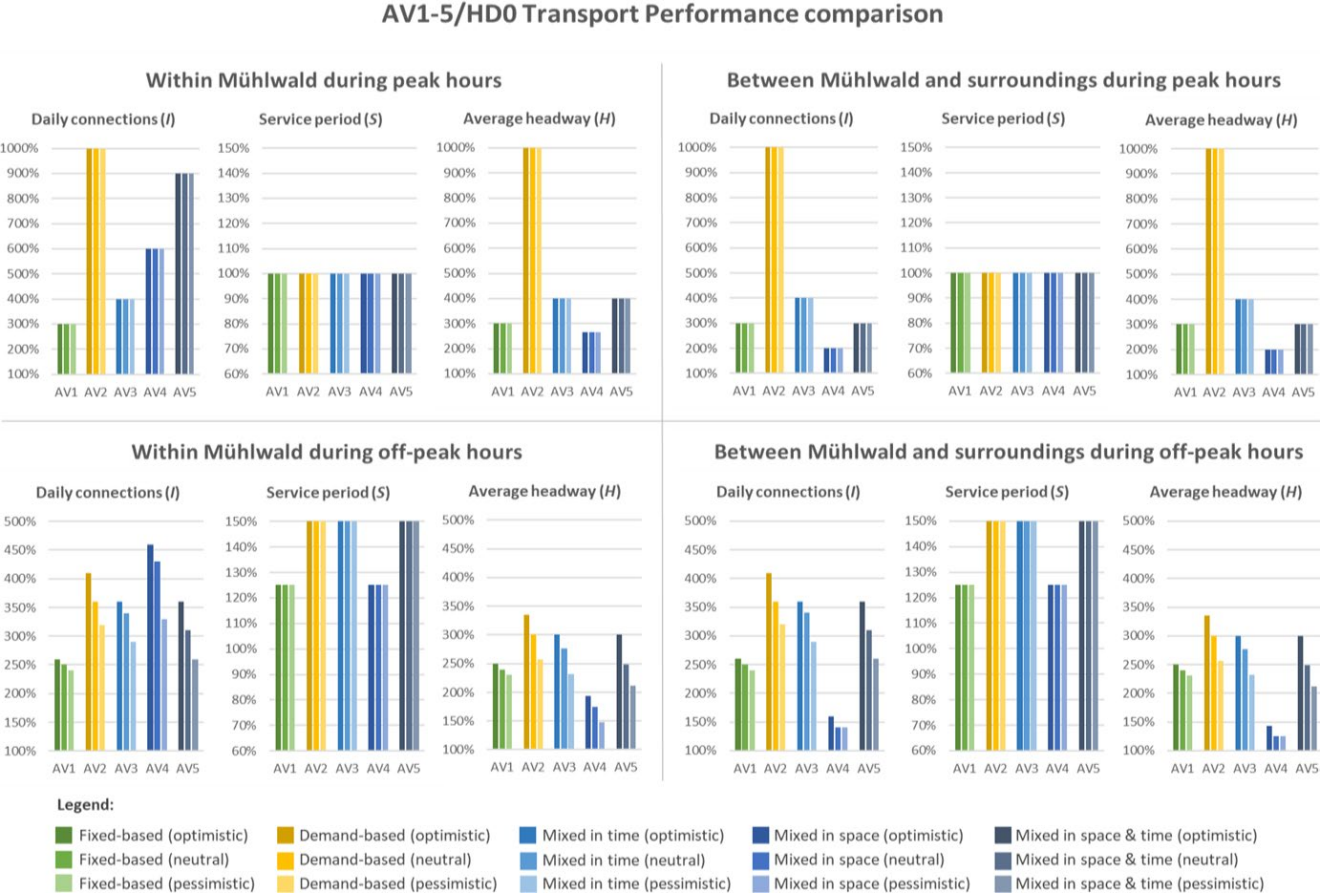
Comparison of the transport performance results of the AV1-5 scenarios against HD0

**Fig. 8:**
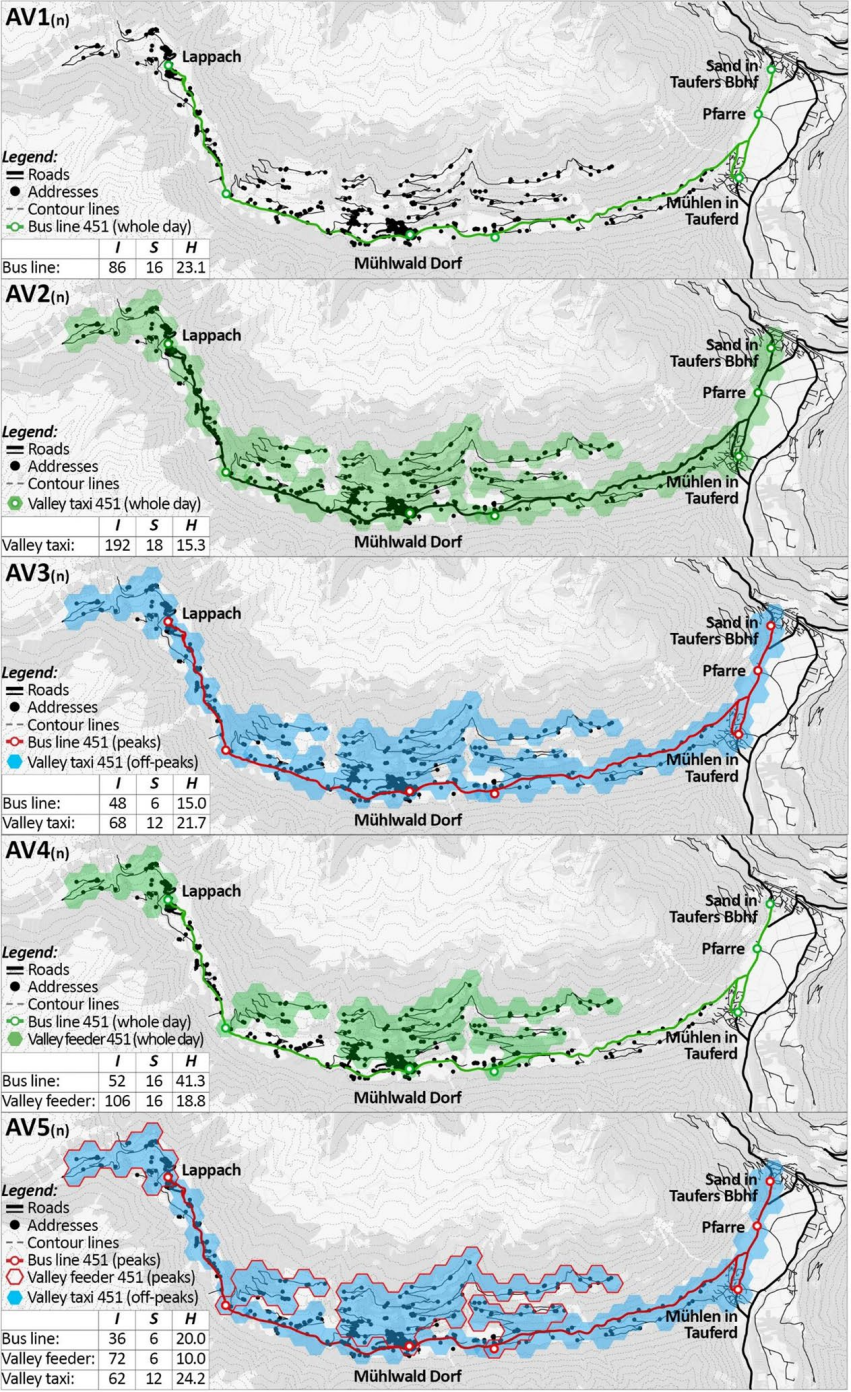
Maps of AV1-5 (neutral variant) with their transport performance results

Figure 7 compares the figures of the transport performance components *I* (daily amount of connections), *S* (service period) and $\bar{H}$ (average headway) of the AV1-5 scenarios against the reference HD0.^16^ The comparison is made for all the three variants (optimistic, neutral and pessimistic). Given the variation of the features of some of the scenarios over space and time, we differentiate the results on a geographical and temporal level. On the one hand, we distinguish between peak and off-peak hours, on the other hand we differentiate between the connections serving the study area (within Mühlwald) and only those linking the study area to its surroundings (between Mühlwald and surroundings). To support the comparison of results, Fig. 8 shows the scenarios AV1-5 (neutral variants) in dedicated maps where the results for *I*, *S* and $\bar{H}$ are summarised.

Within Mühlwald during peak hours: The AV2 scenario sticks out both in terms of connections (120 during the peak hours, i.e. 20/h) and short average headway (6.0 min for all variants). This is linked to the large vehicular fleet that is needed especially in peak hours (8 vehicles). Also, scenarios AV4 and AV5 register a high number of peak-hour connections, ranging between 108 (AV5_(o)_) and 72 (AV4_(p)_). These figures are influenced by the Valley feeder 451, which operates inside of Mühlwald during the peak hours in both scenarios by providing short-distance connections to the main hubs of the bus line 451. As regards the service period, all scenarios are set to serve six peak hours (i.e. 6-9am and 4-7 pm).

Within Mühlwald during off-peak hours: AV2 and AV4 provide the highest amount of connections (64 < *I* < 92 across variants). However, both AV3 and AV5 are more efficient than AV4 in terms of average headway: 20.0 < $\bar{H}$ < 25.8 for AV3, 20.0 < $\bar{H}$ < 28.3 for AV5, and 31.0 < $\bar{H}$ < 40.5 for AV4. Indeed, the deployment of a line-based service only during peak hours in AV3 and AV5 allows higher cost savings used to reinforce the on-demand service during off-peak hours. Conversely, the operation of two kinds of services simultaneously during the whole day (as proposed in AV4) makes costs higher and the average headway less competitive. For all the on-demand services it has to be noticed that, if the demand increased, more connections would be needed, with impacts on the fleet size and duty distance (thus on agency costs). Thanks to the exclusive usage of smaller vehicles operating on demand during off-peak hours, AV2, 3 and 5 allow an extension of the service period up to 20 h (from 6 am until 12 am).

Between Mühlwald and surroundings during peak hours: The number of connections and the average headway is the largest for AV2 (120 connections, one every 6.0 min per direction for all variants). Indeed, all the on-demand connections provided by AV2 are supposed to serve Mühlwald but even connect it to the external hub of Sand in Taufers, according to the current origin–destination patterns of the PT demand. All the other scenarios link Mühlwald to the outside by means of a classic line-based service. The scenarios supplying this line-based service only during peak hours (AV3 and AV5) are those that guarantee the best performances of the line in terms of connections and headway (36 < *I* < 48 and 15 < $\bar{H}$ < 20). Finally, AV1 and AV4 are the least competitive, since they provide an almost homogenous but lower number of connections during the whole day. The service period *S* for the peak hours is six hours in all scenarios.

Between Mühlwald and surroundings during off-peak hours: Since all the connections provided by the Valley taxi serve both Mühlwald and link it to its surroundings, the situation for connections between Mühlwald and surroundings is almost identical to the situation within Mühlwald during off-peak hours for the scenarios AV2, AV3 and AV5. The same applies to AV1, which is based exclusively on a line-based system that constantly serves Mühlwald and its surroundings. The only noticeable difference occurs in scenario AV4. The on-demand Valley feeder only provides connections inside Mühlwald, while the connection to the surrounding areas is covered by the fixed bus line. Since AV4 operates both the bus line and the feeder for the whole day, it guarantees an optimal spatial coverage, which, however, generates higher costs. Because of that, the number of connections and the average headway that may be guaranteed by the bus line during off-peak hours is relatively close to the status quo: 28 < *I* < 32 (against 20 for HD0); and 42 < $\bar{H}$ < 48 (against 60 for HD0).

## Discussion

### Discussion of the results and their policy implications

According to Figs. 7 and 8, AV2 achieves the highest transport performance improvements generally, while keeping the same budgetary restrictions as of now. Indeed, by tailoring the supply to the demand, unnecessary vehicle runs with empty vehicles can be held at a very small number (especially during off-peak hours), reducing the distance-related cost. However, this condition implies that any increase in the demand during the off-peak and peak hours (e.g. because of a modal shift) would require a higher supply and therefore higher costs, which would exceed today’s standards. If one focuses on the cost-effectiveness of the system rather than on just its costs, this situation could be acceptable since revenues would be higher. However, this opens a wider discussion regarding the fare systems and standards. In particular, if a much higher demand was assumed, would the system be able to reach acceptable cost effectiveness by charging users with todays’ fares? Or is it necessary to rearrange the fare system? This question is out of the scope of this paper, but it is a relevant point for future studies intended to deepen the potential replacement of rural bus lines with on-demand services. Another relevant issue of AV2 regards the oversupply (in terms of a vehicular fleet) during off-peak hours. A high number of vehicles (8) is needed during peak hours to meet the demand. Nonetheless, this fleet size is not necessary during off-peak hours, when on average two or three vehicles would be sufficient. Although this oversupply does not result in higher distance-based costs during off-peaks, total agency costs could increase when factoring in parking costs for unused vehicles. If parking costs exceed a certain threshold, keeping the vehicles in traffic could be a more cost-effective alternative, which would increase distance-based costs. Various policies could address this issue and are worth to be considered in future works. For example, regulations increasing as much as possible the amount of passengers per ride during peak hours could allow for a smaller fleet size. Yet, a systemic approach in which the presented scenarios would be introduced on a greater scale over multiple municipalities and regions could be tested. Unused vehicles could serve travel requests in other areas, especially during timeframes when they are underused in their origin location. Finally, the performances calculated for AV2 are based on demand data assuming a homogenous demand distribution within each service hour and a variation across service hours. This implies that the waiting time experienced by users when calling for an on-demand service might vary depending on how the travel requests are temporally distributed. More refined demand simulations are needed to test how waiting time may vary in on-demand services like AV2.

After AV2, the other two scenarios showing the most promising results are AV3 and AV5. Their key feature is their sensitivity to peak/off-peak-hour demand variations. Both scenarios provide a backbone bus line during the peak hours, while they adopt a system of valley taxis during the off-peaks. This flexibility allows for a more tailored management of resources, with positive effects on transport performances. For instance, the average headway of the bus line 451 during peak hours is in the range 15.0–20.0 min for AV3 and AV5, while it is in the range 22.5–41.3 min for AV1 and AV4. This suggests that varying the type of service between peak and off-peak hours may be a cost-efficient solution for AV scenarios, although it has been rarely considered so far in previous studies focused on rural transport automation. Besides being more efficient for the users, this approach allows saving resources and reducing the externalities linked to the off-peak oversupply: empty bus runs are reduced, and their costs and emissions are saved. However, scenarios AV3 and AV5 might have a disadvantage that deserves to be studied: they could be little intuitive for the users. Indeed, the service is not standardised and constant (e.g. one bus every 10 min from 06:00 until 20:00) but is subject to spatial and temporal variations. This could be perceived as too complicated by the users. Even in this field, further research is needed to understand how such a kind of system can be implemented in a user-friendly manner.

### Limits of the study

The main limits of this study regard the following domains: (1) cost calculation, (2) budgeting approach, (3) expected demand reaction, (4) real-world application, and (5) potential methodological improvements.

1. Cost calculation

- *Infrastructure and capital costs*: These two components are not integrated into *AC*, apart for the kilometric depreciation reflecting the vehicle acquisition. However, significant infrastructural and capital expenses will be necessary for AVs, such as the acquisition, instalment and maintenance of sensors, exhaustive 5G networks, and structural road improvements. Nevertheless, these large-scale interventions regard the municipal or state level, thus they do not directly influence the transport providers. For this reason, they are taken as a prerequisite in this study.
- *Unitary cost variations*: We have considered different unitary cost values for AVs for the optimistic, neutral and pessimistic variants. However, the unitary costs could even change across scenario typologies. For example, an increase in fleet size might positively affect insurance costs thanks to large-fleet discounts. At the same time, it could trigger new parking fee policies since the number of vehicles running and parking on public streets increases. To allow a better comparison among scenarios, these relations among cost factors are excluded and we are limited to considering the optimistic, neutral and pessimistic variants.

(2) Budgeting approach

- *Status-quo budget as a benchmark*: The scenarios are elaborated by keeping the same budget as of today. This rationale has two main limitations. First, we do not take into account a potential increase in revenues, which could justify higher investments (as in the study by Imhof et al. 2020). Second, by complying with current budgetary limitations, the thereby formulated scenarios cannot point out additional benefits that could be achieved with an increased budget. Despite these weaknesses, the proposed approach has the advantage of being straightforward for policymakers and avoiding uncertain assumptions/forecasts affecting revenues.
- *Reinvestment of budget savings*: In our work, we have reinvested the *AC* savings derived from automation to increase the performances of the observed line. However, part of the savings derived from transport automation could be used differently. For instance, they could be systematically redistributed among other lines in order to reach higher network competitiveness. Yet, they could be used to make the fares lower for the users. Other works could be done in the future to investigate these options and identify the most suitable combinations.

(3) Expected demand reaction
  We do not consider possible demand changes triggered by the scenarios. However, improved transport performances may lead to a modal shift. This, in return, could constitute a need for the scenarios to increase the overall supply and costs. However, demand forecasts for AVs rely heavily on uncertain assumptions based on stated preferences regarding a technology that is still in an experimental phase (e.g. Chng and Cheah 2020; Fraedrich and Lenz 2016; Hinderer et al. 2018). Although a positive outlook on the willingness to use AVs can be expected (especially for the young generations; e.g. Lee et al. 2017), concrete assumptions about changes in the PT demand cannot be made easily, especially when also taking the attractiveness of private AVs into account. For this reason, this paper has adopted a conservative approach by focusing on the current demand and by leaving aside the possible demand reaction, which is a research topic that deserves specific studies (such as Basu et al. 2018; Becherer and Karduck 2021; Nahmias-Biran et al. 2019).

(4) Real-world application
  As highlighted in the previous point, AVs are not supposed to be the strongly diffused in the transport system before decades (see e.g. Weigl et al. 2022). This could particularly apply to rural areas, which are expected to experience stronger technical obstacles (linked to e.g. road mapping and 5G connectivity; e.g. Neef 2018). Therefore, the results presented in this study have to be interpreted with the proper degree of uncertainty. Nevertheless, exploring the potential of this technology even in the rural context is a valuable point of the international research agenda on AVs (Soteropoulos et al. 2019), as well as an increasingly relevant goal for pilot tests (e.g. Apolitical 2018; Rehrl and Zankl 2018; Riener et al. 2020).

(5) Potential methodological improvements
  Our method allows introducing performance improvements in the scenarios while keeping the today´s agency cost budget and peak-hour capacity as constraints. However, no innovative optimisation framework based on the modelling of an objective function has been developed for this study (e.g. Bazaluk et al. 2021; Ceder 2001). This is a methodological gap that could be addressed in the future, in order to improve the computation and allow, e.g., the extension of this work to broader study areas and systems (such as the whole South Tyrolean bus network). Nevertheless, it has to be mentioned that such methodological innovation was not the goal of this research, which is focused on the systemic development of different scenario typologies and variants for rural public transport automation.

## Conclusions

Despite the limits described above, this study contributes to the understanding of the potential deployments of AVs for the upgrade of the rural PT. For this purpose, we have adopted a policy-oriented and cautious approach, by attempting to understand which improvements could be achieved just by reinvesting the cost savings that the transport automation process could provide. By focusing on a singular PT line to show our approach, we aim to make the underlying concepts as schematic and prototypical as possible. This basic approach can be adapted for enhanced scenarios including a multitude of lines and transport modes in the same or even compatible systems. Although this approach has some limitations as discussed above, it is also straightforward for practitioners and policy makers and it allows avoiding an uncertain discussion about the reaction of the PT demand to the introduction of AVs in the rural context. Additionally, we have not just tested either line-based or on-demand scenarios of rural transport automation (AV1 and AV2), but we have even combined these two types of services over time and space in different manners (AV3-5). This has allowed pointing out how the benefits of different service types can be combined in order to get the best out of both and address the transport challenges of rural areas (i.e. the high time-based demand fluctuation and the remoteness of locations out of reach of the current PT system). This is reflected, e.g., by the results of the AV5 scenario (*mixed in space & time*). Compared to the status quo, the neutral variant of AV5 provides three times more connections between Mühlwald and Sand in Taufers during the peak hours (36 vs 12 con/d) with an average headway of 20.0 min against 60.0 min at the status quo. Additionally, it allows all the remote locations currently out of reach to be connected with the major bus line through a system of on-demand valley feeders, which guarantees an average headway of 10.0 min considering the current PT demand. Finally, a system of on-demand valley taxis operates during the off-peak hours. Based on the current demand, an average headway of about 24.2 min can be guaranteed by this system for a period of 12 h (against 60 min for 10 h at the status quo).

Although these results are specific for our study area, our scenario typologies can be adjusted and applied to other contexts, making the work presented in this paper generalizable. For instance, within the context of the research project “RAAV: Rural Accessibility & Automated Vehicles” (Eurac Research 2022; FWF Project Finder 2021) these scenario typologies are tested in another rural area in Austria, namely the municipality of Sooß in the vicinity of Vienna. Although this area presents different specificities especially in terms of proximity to two major railway stations and to a metropolitan centre, the five scenario typologies have been successfully adapted and tested. Even in this case, the results indicate that the most cost-effective option is the demand-based taxi service (AV2). Secondly, the mixed-in-space scenario (AV4) shows promising results, allowing frequent and constant connections between Sooß and the two reference railway stations. The full results of the test in Sooß are available in the repository of the Vienna University of Technology (TU Wien 2022). The supply scenarios designed in this paper represent not only the output of this work, but also the starting point for a range of evaluations on the impacts of AVs. For instance, they could be used to analyse variations in the users´ (generalised) cost of transport, study the reaction of the demand and changes in the rural mobility patterns, or even investigate their impacts on place-based and person-based accessibility (Dianin et al. 2021, 2022a).

Regardless of these possible developments, this study has attempted to broaden the knowledge on the possible usages of AVs in rural PT, which is still an underexplored topic especially in comparison with urban areas. This context of application deserves increasing attention and insights, given the dependency of several rural areas from private cars and the role AVs might play in the future in changing this paradigm.

## Figures and Tables

**Annex 2:**
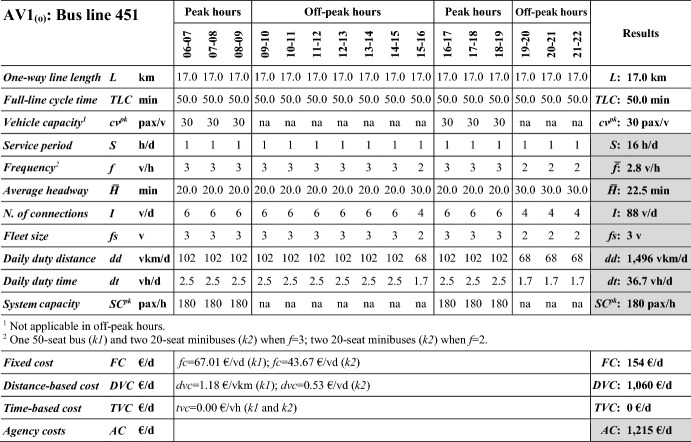
Exemplificative computation of *TP*, *AC* and *SC* for a line-based service included in one of the scenarios (Bus line 451 in AV1_(o)_)

The *TP*, *AC* and *SC* results are highlighted in grey

**Annex 3:**
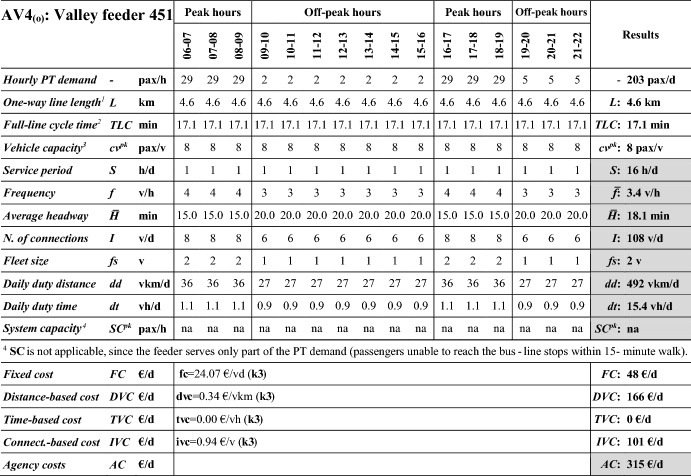
Exemplificative computation of TP, AC and SC for an on-demand service included in one of the scenarios (Valley feeder 451 in AV4_(o)_)

The *TP*, *AC* and *SC* results are highlighted in grey

**Annex 4:**
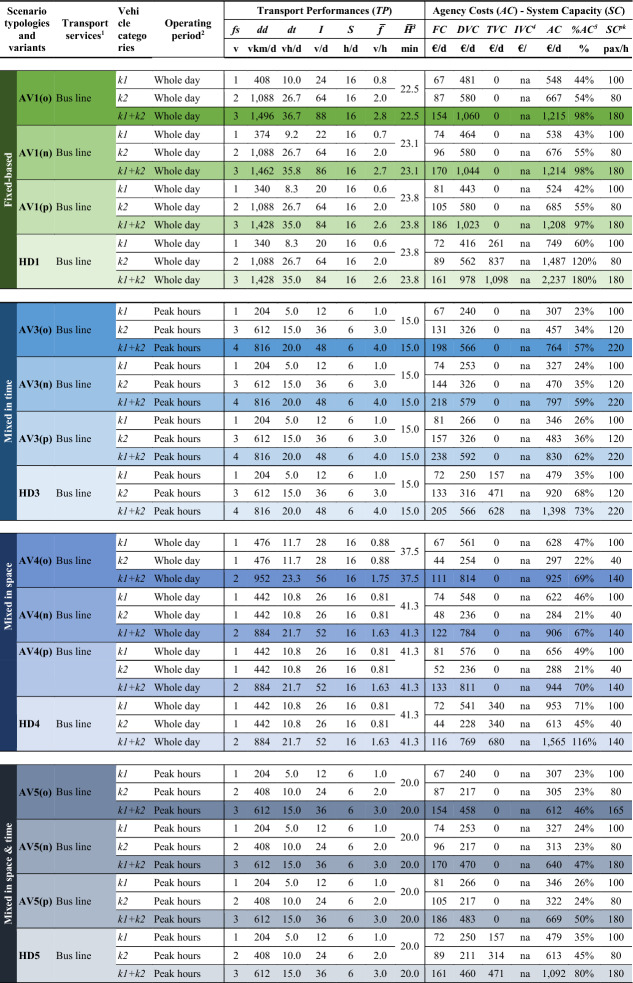
Breakdown of the results presented in Table 5 by vehicle category for the bus line 451 introduced in the scenarios AV1, 3, 4 and 5

^1^Transport services: See Fig. 4

^2^Operating period: Whole day: 6 am - 10 pm (AV1 and AV4); Peak hours: 6 - 9 am and 4 - 7 pm (AV3 and 5)

^3^$\bar{H}$: The headway is not reported for the single vehicle category, since it is not representative of the service provided to the user

^4^*IVC*: Not applicable (na) to the line-based services

^5^***%****AC*: The values reported in this column are obtained from the comparison of *AC* for each vehicle category against the HD0 scenario

## Data Availability

Input and output data used and generated by this research is freely available in the TU Wien Repository at 10.48436/h0mwh-grx90. The dataset includes the case study of Mühlwald (South Tyrol) and Sooß (Lower Austria), both part of the research project RAAV [I 5224-G Internationale Projekte] financed by FWF a the Autonomous Province of Bozen/Bolzano.

## Annexes

**Annex 1:** Details regarding the estimation of the unitary cost components at the status quo (HD0)

Unitary cost components		Unit of measure	HD scenarios		
			*k1* (50-seat bus)	*k2* (20-seat minibus)	*k3* (8-seat van)
Interests	*int*	€/vd	Interest rate of 5.5% and a three-year credit period (with a purchase price of €280,000). Average value from Italian market research (Facile.it n.d.; ASSTRA n.d.)	Interest rate of 7.1% and a three-year credit period (with a purchase price of €60,000). Average value from Italian market research (Facile.it n.d.; ASSTRA n.d.)	Interest rate of 10.3% and a three-year credit period (with a purchase price of €35,000). Average value from Italian market research (Facile.it n.d.; ASSTRA n.d.)
Insurance	*ins*	€/vd	Average insurance premium of €3,720/year for Italian transport agencies (ASSTRA n.d.)	Average insurance premium of €1,015/year for Italian transport agencies (ASSTRA n.d.)	Average insurance premium of €800/year for Italian transport agencies (PCA 2021)
Taxes and levy	*tax*	€/vd	Annual average amount of €2000 (examples from Bösch et al. 2018)	Annual average amount of €1000 (examples from Bösch et al. 2018)	Annual average amount of €600 (examples from Bösch et al. 2018)
Parking^*a*^	*prk*	€/vd	na	na	na
Highway Tolls^*a*^	*tls*	€/vd	na	na	na
Overheads	*ovr*	€/vd	Overheads may vary significantly depending on the size of the fleet managed by the transport agency (ASSTRA 2013; Bösch et al. 2018). Based on an internal consultation with the South Tyrolean transport agency, a value of 17.37€/vehicle-day has been estimated considering the daily administrative, management and communication personnel costs and the total fleet managed. The result is generally consistent with the examples from Bösch et al. (2018): 14 CHF/vehicle-day		
Cleaning (for line-based services)^*b*^	*cln*_*(lb)*_	€/vd	One cleaning service of €50 every second day (internal consultation with the South Tyrolean transport agency)	One cleaning service of €35 every second day (internal consultation with the South Tyrolean transport agency)	na
Depreciation	*dpr*	€/vkm	Function of a purchase cost of €280,000 and a vehicular life of 450,000 service km (internal consultation with the South Tyrolean transport agency)	Function of a purchase cost of €60,000 and a vehicular life of 300,000 service km (examples from Bösch et al. 2018; market research in Italy)	Function of a purchase cost of €35,000 and a vehicular life of 300,000 service km (examples from Bösch et al. 2018; market research in Italy)
Fuel	*ful*	€/vkm	Fuel consumption of 3.5 km/l and fuel price of €1.50/l before the Covid-19 and Ukraine crisis (market research in Italy)	Fuel consumption of 7 km/l and fuel price of €1.50/l before the Covid-19 and Ukraine crisis (market research in Italy)	Fuel consumption of 14 km/l and fuel price of €1.50/l before the Covid-19 and Ukraine crisis (market research in Italy)
Tyres and lubricants	*tyr*	€/vkm	Tyres: €250 for the purchase of each tyre; 4 tyres/vehicle for all the vehicle categories; 60,000 km of average tyre life. This leads to €0.0167/km (ASSTRA 2013)		
			Lubricants: €0.0045/g for the purchase of lubricants; consumption of 1.25 g/km for all the vehicle categories. This leads to €0.0056/km (ASSTRA 2013)		
Maintenance	*mnt*	€/vkm	Value of €0.15/km provided from ASSTRA (2013)	Assumed value of €0.08/km by rescaling the data from ASSTRA (2013) and the examples from Bösch et al. (2018)	Assumed value of €0.08/km by rescaling the data from ASSTRA (2013) and the examples from Bösch et al. (2018)
Crew remuneration	*crw*	€/vh	Data provided by ASSTRA (2013) for an average Italian extra-urban provider considering a unitary annual cost for driving personnel of 35,302 €/year, an average of 250 days of driving/year, and an average of 4.5 h of driving/day (including breaks). The resulting value (31.38 €/vh) has been cross-checked in terms of share of the driver cost on the total daily agency costs. The result indicates a share of 40.5%, in line with the value of 40% provided by INVITALIA (2019) for South Tyrol		
Cleaning (for on-demand services)^*c*^	*cln*_*(od)*_	€/v	na	na	One cleaning service of €25 every 40 trips (Bösch et al. 2018; consultation with the South Tyrolean transport agency)

^a^The transport provider does not supply services along highways and does not pay any parking fee to the regional public authority

^b^*cln*_*(lb)*_ is not applicable to *k3*, since this vehicle category is used in this study only for on-demand services

^c^*cln*_*(od)*_ is not applicable to *k1* and *k2*, since these vehicle categories are used in this study only for line-based services

## Footnotes

1 The cost of the acquisition, distribution, instalment and maintenance of the infrastructure needed for the setup and operation of AV-systems (such as sensors, exhaustive 5G networks, structural measures for road improvements or road signs) is not the focus of our study. Therefore, *AC* includes solely operational expenses covered by the transport provider, as stated by Ricci (2011).

2 The Italian Local Labour System classification clusters municipalities according to data of in- and out-going commuting flows, by defining clusters shaped by interlinked daily commuting relations (ISTAT 2015).

3 Data directly provided by STA to the authors on request (not publicly available). The data includes only the costs for operating the service, while the expected revenues are excluded from this amount.

4 This source provides average values for all the considered cost items for urban and extra-urban bus services based on the empirical analysis of a representative sample of Italian PT agencies. We considered the data for extra-urban bus services for our estimation.

5 Bus line 451 deployed in HD1,3,4,5 and AV1,3,4,5.

6 Valley taxi 451 and Valley feeder 451 deployed in HD2,3,4,5 and AV2,3,4,5.

7 *L* is equal to 17.0 km for the line-based services in the AV and HD scenarios.

8 *TLC* is equal to 50 min for the line-based services in the AV scenarios. It is slightly lower than the HD0 scenario (60 min) since the drivers' breaks at the terminals are assumed to be not necessary anymore with the introduction of AVs.

9 *cv*^*pk*^ varies across scenarios depending on the combinations of vehicle categories that are deployed during the peak hours (see Annex 2 and 4). *cv*^*pk*^ is equal to: 30 pax/v (AV1); 27.5 pax/v (AV3); 35 pax/v (AV4); and 30 pax/v (AV5).

10 Data obtained from the check-in and check-out ticketing system provided to the authors on request. The data has been cross-checked with the overall daily figures provided by annual statistics (ASTAT 2022).

11 72 pax/h in peak hours (6-9 am and 4-7 pm); 5 pax/h in day off-peak hours (9 am-4 pm); 12 pax/h in night off-peak hours (7-10 pm). Most of the demand is headed outside Mühlwald in the timeframe 6 am-4 pm, and vice versa during the timeframe 4-10 pm. This data is used to design the Valley taxi 451. For the Valley feeder 451, we assume a 60% decrease in demand, since only 40% of the residential addresses in Mühlwald are more than a 15-min walk from a stop of the bus line 451.

12 For all the on-demand services, we deploy only *k3* vehicles. Thus, the vehicle capacity is always equal to 8.

13 *L* is equal to 12.6 km for the Valley taxi 451, and 4.6 km for the Valley feeder 451. The former is the average distance between all the residential addresses of Mühlwald and the terminal of Sand in Taufers Bbhf (the farthest point reached by the Valley taxi 451). The latter is the average distance between all the residential addresses of Mühlwald and the closest main stop of the bus line 451 (excluding the addresses that can reach a stop within a 15-min walk). According to Bösch et al. (2018), both distances incorporate a 20% share of extra kilometres for pick-up operations and relocation, maintenance and cleaning.

14 *TLC* is equal to 47.3 min for the Valley taxi 451, and 17.1 min for the Valley feeder 451. *TLC* is obtained based on *L* and by assuming a commercial speed of 32 km/h, according to the assumptions from Bösch et al. (2018) about on-demand AVs.

15 *cv*^*pk*^ is always equal to 8 pax/v, since only *k3* vehicles are used for the Valley taxi and Valley feeder.

16 For the sake of consistency, the $\bar{H}$ results have been reversed: $\bar{H}$ values > 100% show to which extent $\bar{H}$_*AVn*_ is lower than $\bar{H}$_*HD0*_.
